# Death Secondary to Brain Herniation in Classic MSUD Patients: A Case Series From One Center

**DOI:** 10.1002/jmd2.70135

**Published:** 2026-11-13

**Authors:** Maria C. Laso‐Salazar, Debra S. Regier, Kimberly A. Chapman, Samantha Vergano, Nadia Falah, Jennifer L. Harmon, Nina Mickle, Natasha E. Shur, Christina L. Grant, Erin MacLeod, Danielle Starin, Jamie L. Fraser, Eyby Leon

**Affiliations:** ^1^ Universidad Científica del Sur Lima Peru; ^2^ Atrium Health Levine Children's Specialty Center Charlotte North Carolina USA; ^3^ Children's Hospital Los Angeles Los Angeles California USA; ^4^ Division of Genetic Medicine Seattle Children's Hospital Seattle Washington USA; ^5^ MD Genetic Clinic Bradenton Florida USA; ^6^ Department of Neurology Atrium Health Wake Forest Baptist Hospital Winston‐Salem North Carolina USA; ^7^ Rare Disease Institute, Children's National Hospital Washington DC USA; ^8^ Division of Genetics, Department of Pediatrics University of Virginia School of Medicine Charlottesville Virginia USA

**Keywords:** amino acid metabolism, brain herniation, leucine, maple syrup urine disease

## Abstract

Maple Syrup Urine Disease (MSUD) is an inborn error of metabolism associated with defects in the branched‐chain alpha‐keto acid dehydrogenase complex. Despite careful management, patients may experience metabolic decompensation, elevation of leucine levels, cerebral edema, brain herniation, and death. Two populations have been reported to be the most affected: Mennonite and Ashkenazi Jewish. There are three genes associated with this condition, with *DBT* being the least frequently involved and usually associated with milder or thiamine‐responsive MSUD. We present four patients, three of Salvadoran and one of Afghan ancestry, diagnosed with classic MSUD who developed cerebral edema, herniated, and died despite aggressive medical management. All patients had pathogenic variants in the *DBT* gene and were not thiamine‐responsive. They all presented with elevated plasma leucine levels that were not their highest recorded after diagnosis. Decompensation in an MSUD patient should be approached with extreme caution, considering that brain edema can progress very rapidly to brain herniation and death, especially since there are no reliable biochemical markers that can predict this fatal outcome. The phenotype and genotype of classic MSUD need to be described more thoroughly, since they can have a more severe presentation and higher mortality rate than previously reported and might be misleading to the treating clinician when genetic testing results are of uncertain significance. Our cohort presents four patients of non‐Mennonite or Ashkenazi Jewish descent with fatal brain herniation occurring rapidly without being preceded by their highest plasma leucine peak registered, significant ketonuria, elevated anion gap, and/or hyponatremia on admission. This highlights the importance of studying different populations affected by MSUD and considering liver transplantation as the potential best treatment option for certain patients.

## Highlights

- Plasma leucine levels during a brain herniation event are not necessarily the highest levels recorded on patients affected with classic MSUD.
- Biomarkers like plasma leucine levels, ketosis, hyponatremia, and elevated anion gap, lack a predictive threshold on admission for imminent cerebral edema and brain herniation in patients with classic MSUD.
- All the patients reported in this series had pathogenic variants in the DBT gene, were not thiamine‐responsive, and represent the predominant genotype in our Central American MSUD patients.

## 1 Introduction

Maple Syrup Urine Disease (MSUD) is an inherited disorder of branched‐chain amino acid (BCAA) metabolism caused by deficiency of the branched‐chain alpha‐ketoacid dehydrogenase complex (BCKDC). This complex catabolizes leucine, isoleucine, and valine, which are essential amino acids that are required for energy production and protein synthesis through the mammalian target of rapamycin (mTOR) signaling pathway and stimulates neurotransmitter synthesis in the brain [1].

This condition is caused by biallelic pathogenic variants in one of three genes: *BCKDHA* in 45% of cases, *BCKDHB* in 35%, or *DBT* in 20% [2]. There are four different clinical types of MSUD: classic, intermediate, intermittent, and thiamine responsive [2]. The classic phenotype presents neonatally and is the most severe, characterized by irritability, lethargy, and focal dystonia, with elevated BCAA levels, high anion gap metabolic acidosis, ketosis, and/or hyponatremia [2]. Classic MSUD patients are mainly treated with a lifelong restricted diet consisting of low intake of BCAAs [3]. Additionally, orthotopic liver transplantation is increasingly considered an effective therapeutic strategy for this condition, restoring hepatic BCKDH activity and BCAA homeostasis [4].

Studies have been conducted in some specific population groups, such as the Ashkenazi Jewish population and the Old Order Mennonites, which have a high carrier frequency of MSUD‐related variants in *BCKDHB* and *BCKDHA* respectively [5]. According to Strauss et al., 37% of 184 Mennonite patients with the diagnosis of MSUD who were born between 1963 and 1988 died of complications of metabolic encephalopathy. For patients born after the year 1988, mortality was lower due to the integration of metabolic services in primary care, improved access to amino acid tests, and local availability of total parenteral nutrition (TPN) for MSUD. However, 1.4% died of complications of cerebral edema [6].

The Rare Disease Institute at Children's National Hospital has evaluated a total of 39 MSUD patients since 2010. Over 60% or 24 of our patients are from Central/Latin America, and almost all of them have a classic presentation due to pathogenic variants in the *DBT* gene. The majority of patients, 15 in total (62.5%), have undergone liver transplantation. In this paper, we describe the MSUD patients who died due to brain herniation in our center since 2010. Interestingly, all of them are from underrepresented populations and did not undergo liver transplantation.

## 2 Methods

The methodology consisted of a descriptive retrospective chart review of all the MSUD patients who were evaluated at Children's National Hospital and died due to brain herniation from 2010 to 2025.

The average leucine level during the stable period was calculated using levels obtained in a 6‐month period. Classic MSUD was defined as neonatal presentations of MSUD‐related symptoms.

In this study, we consider the definition of underrepresented populations as a subgroup of the population, identified by race and ancestry, whose representation is low in genetic studies, compared to the general population, resulting in a lack of genetic information [7].

## 3 Case Series

Patient 1 was a girl of Afghan ancestry who was diagnosed after positive newborn screening. Her MSUD was well controlled until age 2, when she began vomiting her metabolic formula requiring nasogastric (NG) tube placement. She was transferred to our center after being admitted for 1 month due to a 3‐month history of poor oral tolerance for further management and potential gastrostomy tube placement. She was receiving her metabolic formula, isoleucine, valine, along with BCAA‐free TPN. Her NG feeds were stopped due to persistent vomiting during her first day of admission in our center, when she was found to have altered mental status, low tone in extremities, and recurrent episodes of emesis despite antiemetic therapy with ondansetron. Her initial leucine level on admission was 569 μmol/L, plasma beta‐hydroxybutyrate was negative, she had a normal anion gap, and her sodium level was 137 mmol/L. Overnight, she became unresponsive, requiring intubation due to worsening of her mental status and abnormal movements. Her sodium was 131 mmol/L, and a head computed tomography (CT) scan in less than 12 h from admission revealed severe diffuse vasogenic edema. Hypertonic fluids were started along with increased caloric supply via continuous enteral feeds, more concentrated intravenous (IV) dextrose, and BCAA‐free TPN. Her plasma leucine level 12 h after admission was 735 μmol/L and sodium went up to 138 mmol/L. A second head CT five hours after the initial one showed improvement of cerebral edema and repeat plasma leucine level went down to 527 μmol/L. However, a repeat CT on her third day of admission showed progression of infratentorial brain edema with near total compression of subarachnoid spaces with caudal displacement of the cerebellum despite improvement of her plasma leucine level to 406 μmol/L and a sodium level of 141 mmol/L. Subsequently, the patient was declared brain dead and extubated.

Patient 2 was a 20‐year‐old woman of Salvadoran ancestry diagnosed by newborn screening; previously reported by Billington et al. as Case 8 [8]. She had a history of arrhythmia treated with ablation, migraines without aura, anxiety disorder, Attention‐Deficit/Hyperactivity Disorder (ADHD), asthma, chronic constipation, and abdominal pain. She had a history of over 40 hospitalizations, the most recent of which were due to migraine headaches and abdominal pain. She became pregnant at 18 years of age and carried her pregnancy without metabolic decompensations since she was able to adhere to medical management. At 19 years old, she began to have higher leucine levels, due to an inability to tolerate her metabolic formula which required a gastrostomy tube (G‐tube) placement. She was also having trouble preparing her formula recipe, especially after a metabolic decompensation event with her highest leucine level recorded after diagnosis, 1097 μmol/L. She was admitted for headaches, upper respiratory congestion, decreased oral intake, and leakage around her G‐Tube for 1 month. She was placed on dextrose 10% fluids with normal saline at 1.5 maintenance, isoleucine, valine, and intralipids at 3 g/kg/d. Her initial leucine level was 670 μmol/L, sodium level was 138 mmol/L, urine ketones were negative, blood beta‐hydroxybutyrate level was mildly elevated at 0.25 mmol/L, and anion gap was elevated at 22. In less than 12 h she developed altered mental status and became unresponsive, her repeat sodium was 131 mmol/L, and her head CT scan showed mild diffuse parenchymal edema and interval descent of the cerebellar tonsils below the foramen magnum. She was transferred intubated to the Pediatric Intensive Care Unit (PICU) and received hypertonic saline, improving her sodium level to 136 mmol/L in less than 2 h. She was placed on hemodialysis along with BCAA‐free TPN without knowing that her leucine level was 311 μmol/L at that moment. A repeat CT scan 6 h later showed interval decrease in effacement of ventricles, basal cisterns, and cerebellar tonsillar herniation, with a sodium level of 151 mmol/L. Despite these interventions, and a leucine of 176 μmol/L, her cerebral edema worsened with a retroclival hemorrhage, and ultimately, she died.

Patient 3 was a 13‐year‐old boy of Salvadoran ancestry diagnosed with MSUD after positive newborn screening. He had over 16 hospitalizations at our center before his last admission with us. He was admitted for vomiting, dehydration, headaches, and abdominal pain for 3 days. His sodium on admission was 139 mmol/L; he had an elevated anion gap, and urine ketones were 3+. He was placed on dextrose fluids at 10% with normal saline at 1.5 maintenance, isoleucine, and valine. He became lethargic overnight and experienced a generalized tonic–clonic seizure, followed by tonic posturing. A brain CT scan revealed diffuse cerebral edema and signs of pre‐subtentorial herniation. Repeat sodium level was 140 mmol/L; his plasma leucine level was 738 μmol/L, up from 631 μmol/L on admission, requiring the initiation of dialysis which subsequently decreased the plasma leucine level to 378 μmol/L. Despite treatment and dialysis, the patient was declared brain dead on his third day of hospitalization.

Patient 4, a 25‐year‐old woman, previously reported by Falah et al. [9], of Salvadoran ancestry, was diagnosed with classical MSUD at 11 days of life. She was well‐controlled in our center throughout most of her childhood and adolescence. At 19 years old she had an uncomplicated pregnancy. However, she developed anxiety and panic attacks during her adulthood, and at 24 years old, she was admitted for psychosis, a urinary tract infection and hyperleucinemia, with her highest leucine level reaching 2549 μmol/L. She was managed with IV dextrose, intralipids and protein restriction, reducing leucine levels to 614 μmol/L, until her discharge. During this admission, it was discovered that she had been consuming methylmalonic acidemia (MMA) formula instead of her prescribed MSUD formula. The MMA formula had been shipped erroneously by her formula supplier for at least 1 month. Of note, the MMA formula she had been drinking was rich in leucine. One month after discharge, the patient relocated to a different state and became less responsive to contact from the local metabolic clinic. Five months after discharge, she presented to a local emergency room with hallucinations and dissociative identity after not taking her metabolic formula for 1 day. Her initial leucine level was 1221 μmol/L, with sodium at 142 mmol/L; no ketosis and normal anion gap. She was placed on dextrose fluids and BCAA‐free formula. Her brain CT showed basal ganglia edema with a leucine level of 1075 μmol/L, and sodium levels of 130 mmol/L at the time of deterioration in less than 48 h. Despite treatment with hypertonic saline, her condition deteriorated quickly, showing worsened cerebral edema, resulting in brain death before hemodialysis was performed.

Demographic characteristics, total hospitalizations, *DBT* variants, and plasma leucine levels of all four patients are summarized in Table 1.

**TABLE 1:** Medical aspects of each patient.

	Patient 1	Patient 2	Patient 3	Patient 4
Ethnicity	Afghan	Salvadoran	Salvadoran	Salvadoran
Sex	Female	Female	Male	Female
Age at death in years	2	20	13	25
Number of hospitalizations	5	43	16	17
*DBT* variant(s)	Homozygous c.853A > C (p.Thr285Pro)	c.916 T > C (p.Ser306Pro) + delEx2	Homozygous delEx2	c.916 T > C (p.Ser306Pro) + delEx2
ACMG variant classification [10]	VUS	Pathogenic	Pathogenic	Pathogenic
Leucine levels at diagnosis (μmol/L)	2811	1336	1179	132
Average leucine levels during a stable period (μmol/L)	177	176	463	233
Highest leucine levels after diagnosis (age)	1385 (2 y/o)	1097 (19 y/o)	1460 (7 y/o)	2549 (24 y/o)
Leucine level on last admission (μmol/L)	569	670	631	1221

Abbreviation: ACMG, American College of Medical Genetics and Genomics.

## 4 Discussion

The main risk in MSUD patients is acute metabolic decompensation, leading to brain edema, herniation, and death. The frequent hospitalizations of some patients in this case series suggest they had difficulty maintaining adequate metabolic control in the outpatient setting and may have already been more vulnerable to a fatal metabolic crisis. In such patients, clinicians should maintain a very low threshold for intensive inpatient management. Two of the four patients (2 and 3) underwent hemofiltration, which reduced their leucine plasma levels within hours, but it did not lead to clinical improvement. It is possible that this intervention does not affect intracranial leucine levels at an advanced stage of metabolic decompensation in a disorder that seems to be more severe in our patient population with classic MSUD due to *DBT* variants as described by Billington et al. with functional studies that showed a decreased amount of protein product in delEx2;c.916 T > C liver cells and absence of protein product in delEx2 homozygous hepatocytes [8]. According to Atwal et al. [11], hemodialysis combined with dietary BCAA enables a fast reduction of leucine levels compared to conservative methods. However, they emphasize that it must be done with caution, considering there is a theoretical risk of exacerbating brain edema due to fluid shifts with reduction of leucine. In all our cases where this procedure was performed, neurological status deteriorated despite careful management.

Myers et al. published a case of a 7‐day‐old baby girl with MSUD of Nicaraguan ancestry detected by newborn screening. She was admitted asymptomatic, with a plasma leucine level of 1980 μmol/L, and started on diet therapy. She began to feed poorly and was noted to be lethargic 12 h after admission, and head CT scan showed diffuse brain swelling. Three hours after receiving intravenous fluids, her lethargy improved, and her repeat plasma leucine level 24 h after admission was 1340 μmol/L [12]. These findings showed that clinical and radiological deterioration can happen even when plasma leucine levels are decreasing at a fast rate, so cerebral edema may be the first sign of decompensation, even if leucine levels appear to be correctly managed. She was discharged, and at her 6‐month follow‐up, she was thriving.

Sulaiman et al. described two MSUD patients with cerebral edema from Saudi Arabia with a history of poor dietary compliance who died after significant management efforts. Patient 3, a 14‐year‐old male homozygous for a missense variant in *BCKDHA*, presented with cerebral edema and a leucine level of 2400 μmol/L. He died on the third day due to brain herniation, with a leucine level of 560 μmol/L after IV fluids, BCAA‐free formula, and continuous renal replacement therapy (CRRT). Similarly, Patient 4, a 15‐year‐old male homozygous for a nonsense variant in the *DBT* gene, was admitted with high plasma leucine levels and diffuse brain edema. Despite reducing plasma leucine level from 2030 to 1033 μmol/L with IV fluids, BCAA‐free formula, and CRRT, and undergoing a decompressive craniotomy, he still progressed to tonsillar herniation and death [13].

Acute metabolic decompensation in MSUD is usually monitored using biomarkers such as plasma leucine levels, ketonuria, and anion‐gap; however, our findings highlight that these biomarkers often lack a predictive threshold on admission for imminent cerebral edema and brain herniation. While acute reductions in sodium and rapid decline in effective serum osmolality are recognized risk factors that promote brain edema, fatal brain herniation in our cohort occurred at variable leucine concentrations, often substantially below each patient's historical peak and without a consistent biochemical pattern. Furthermore, brain herniation progressed even after the correction of hyponatremia or without persistent sodium deficits. This emphasizes that while established laboratory parameters are indispensable for assessing metabolic control, they remain unreliable for predicting which patients will progress to irreversible herniation.

Our institution's protocol includes ordering a quantitative plasma amino acid profile, which can take between 6 to 72 h to return results, depending on the day of admission (weekday vs. weekend). We start dextrose fluids with normal saline at 1.5 to twice maintenance. Continuous insulin is not part of the protocol unless persistent blood glucose levels exceed 200 mg/dL. We also monitor electrolytes, anion gap, and ketones that are repeated daily or more frequently if abnormal. If the patient is found to have an elevated leucine level, we initiate BCAA‐free formula and increase isoleucine and valine doses even through a nasogastric tube and follow plasma amino acids at least daily. If the patient is not able to tolerate oral formula or medications, we start BCAA‐free TPN. If sodium levels are low, we start hypertonic saline along with dextrose fluids. If plasma leucine level is 2000 μmol/L or above, we initiate hemofiltration. The patient is admitted to the ICU if there are signs of altered mental status for neurological checks hourly. We order a rapid head CT if there are signs or concerns for altered mental status or brain edema; this includes headaches.

While there are emergency measures that can lower plasma leucine levels in acute metabolic crises, they may not be able to prevent herniation once severe cerebral edema has been established. Orthotopic liver transplantation provides long‐term metabolic stability, reducing the risk of acute crises [4]. Non‐transplanted patients are more vulnerable to fatal neurological complications during metabolic decompensation.

All the patients reported in this article had variants in the *DBT* gene and were not thiamine‐responsive. Although the *DBT* gene is reported to affect only 20% of the MSUD patients [2], it represents the predominant genotype in our Central American cohort. Billington et al. describe the possibility of a founder effect in the Central American population for two *DBT* variants, delEx2 and c.916 T > C. They investigated this possibility by testing the surrounding region of the *DBT* gene, finding an area of homozygosity of 16.7 Mb, suggesting identity by descent for the studied alleles [8]. They also mention that, considering the large number of immigrants from Central America in Washington DC, the carrier rate for the variants delEx2 and c.916 T > C, found in our patients 2 and 4, may be estimated at 1 in 94 persons with this ancestry [8].

It is important to highlight that, with the exception of Patient 3, the highest leucine levels recorded occurred close in timing to the fatal event. Recurrent severe peaks might indicate increased vulnerability to acute complications. It is possible that these recent elevations could help identify patients at higher risk, but given our small sample size, larger studies are needed to confirm this observation.

## 5 Conclusions

Brain herniation remains one of the most devastating complications of MSUD, which needs urgent attention and improved strategies for its prevention and management considering this could happen even if the patient has been able to tolerate higher leucine levels in prior admissions. Our cases and some reported in the literature show that plasma leucine levels during a brain herniation event are not necessarily the highest levels recorded after diagnosis and may be significantly lower than those previously recorded.

We present four patients from underrepresented populations who died due to brain herniation as a consequence of MSUD caused by pathogenic variants in the *DBT* gene. We aim to highlight the importance of studying not only these populations, but also all patients affected by classic MSUD, to underscore the importance of careful lifelong clinical management that may be more challenging for some patients.

Reporting more cases like these in the scientific literature is essential to identify critical factors that may influence brain herniation. This can deepen our understanding of the pathophysiology of brain herniation in metabolic decompensation and establish tailored interventions. Future research should expand access to genetic testing in underrepresented populations and explore different therapeutic approaches in cases like these to prevent fatal outcomes.

## Author Contributions

**Maria C. Laso‐Salazar:** design, writing, clinical data collection. **Debra S. Regier:** patient management and clinical follow‐up. **Kimberly A. Chapman:** patient management and clinical follow‐up. **Samantha Vergano:** patient management and clinical follow‐up. **Nadia Falah:** patient management and clinical follow‐up. **Jennifer L. Harmon:** patient management and clinical follow‐up. **Nina Mickle:** patient management and clinical follow‐up. **Natasha E. Shur:** patient management and clinical follow‐up. **Christina L. Grant:** patient management and clinical follow‐up. **Erin MacLeod:** patient management and clinical follow‐up. **Danielle Starin:** patient management and clinical follow‐up. **Jamie L. Fraser:** patient management and clinical follow‐up. **Eyby Leon:** conceptualization, design, supervision and validation, review and editing, patient management and clinical follow‐up.

## Funding

The authors have nothing to report.

## Ethics Statement

This study was conducted in accordance with the Declaration of Helsinki. All clinical data were collected retrospectively and fully anonymized, and there is no identifiable patient information included; therefore, ethics committee approval was not required according to local institutional guidelines.

## Consent

Patient consent was not required for this study as it is a retrospective review of fully de‐identified clinical and biochemical data. All identifiers were removed to ensure patient anonymity.

## Conflicts of Interest

The authors declare no conflicts of interest.

## Data Availability

The data that supports the findings of this study are available on request from the corresponding author.
